# Antiamoebic Chemoprophylaxis Using Quinfamide in Children: A Comparative Study

**DOI:** 10.1100/tsw.2002.174

**Published:** 2002-04-20

**Authors:** Nicolas Padilla, Rosalinda Diaz, Alfonso Alarcon, Roberto Barreda

**Affiliations:** Unidad de Investigacion, Escuela de Enfermeria y Obstetricia de Celaya, Universidad de Guanajuato, Searle de Mexico, S.A. de C.V., Mexico; Dirección Médica, Searle de Mexico, S.A. de C.V., Mexico

**Keywords:** chemoprophylaxis, quinfamide, amebiasis

## Abstract

This study sought to examine whether the administration of quinfamide at 3- or 6-month intervals diminished the frequency of *Entamoeba histolytica* cysts in stool samples compared to controls. The prospective, longitudinal, randomized, single-blind study examined children from six primary schools in Celaya and Neutla, Guanajuato. Of the 1,524 students in these schools, we selected participants for the study as follows: Children were included in the study if their parents agreed in writing to the study and if the children demonstrated evidence of *E. histolytica* cysts after a parasitoscopic analysis by concentration (PSC) in three samples over consecutive days using Faust’s method. Those included in the study received a single 4.3-g/kg dose of quinfamide, and we performed PSC on days 5, 6, and 7 following dose administration to examine whether quinfamide had affected the presence of the cysts. The study participants who tested negative for cysts were divided into three groups: Group 1 had 102 patients who underwent quinfamide treatment and three CPS analyses after the 12 months of the study; Group 2 had 98 subjects who underwent the quinfamide treatment and three CPS analyses at months 3, 6, 9, and 12 after their entrance into the study; and Group 3 had 102 patients, who underwent the quinfamide treatment and series of three CPS analyses at months 6 and 12 of the study. All participants received the dose of quinfamide after providing stool samples and after a clinical gastrointestinal history was obtained. Further clinical gastrointestinal data were collected 5 days after the quintamide dose was administered. We used EpiInfo 6.0 for statistical analysis, calculating *X*^2^ and *p* values for the clinical data and the CPS data after the 12 months concluded. Of the initial samples of 1,524 subjects, 308 (20.2%) had Entamoebic cysts. Of these, six were further eliminated because they did not meet the inclusion requirements. At the conclusion of the study, Group 1 presented with 37.6% of subjects still testing positive for cysts; of Group 2, 12.5% tested positive; and in Group 3, 23.5% of participants tested positive for cysts (*X*^2^ = 16.8; df = 2; *p* = 0.0002). For comparisons of groups 1 and 2 and 1 and 3, *p* > 0.05. We conclude that antiamoebic chemoprophylaxis can be a choice for control of amoebic infection where personal hygiene and food consumption habits are not improving.

## INTRODUCTION

The World Health Organization's committee of experts on amoebic infection defines amoebic infection as a situation in which *Entamoeba histolytica* is carried in the organism, even in the absence of clinical symptoms[1]. Amoebic infection has a worldwide distribution, but it is most frequent in poor communities and in areas with unhealthy environmental conditions[2]. In Mexico, it is thought that 27% of the population has an amoebic infection[3]; of this percentage, 20% are carriers, 2% are ill, 5.9% test positive in blood tests, and of the sick, between 0.1 and 0.2% die[4]. In 2000, more than 1.3 million cases of symptomatic intestinal amoebic infection and 6,269 cases of liver abscess because of amoebic infection were reported[5]; thus, it is reasonable to assume that in 2000, there were around 13 million carriers who were an important source of the spread of infection.

The primary strategy for controlling amoebic infection should focus on preventing the spread of the infection through fecal-oral contact. Such preventions include washing hands before eating and after defecation, and appropriate food preparation practices. In the majority of cases, transmission occurs because of ingestion of food that was prepared by people who carry the infection, but are asymptomatic[6,7]. This observation was demonstrated in Costa Rica, where the prevalence of amoebic infection ranges from 6 to 17% in six areas of the country, but there is no relationship between the value of a home or the presence of sanitary facilities and the frequency of amoebic infection[4].

The second line of defense is the treatment of symptomatic cases and the management of carriers. To plan effective amoebic infection control programs that are based on chemiprophylaxis, medications that are effective against *E. histolytica* in the intestinal lumen are necessary[6,8]. Antiamoebic medications that require multiple doses and many days of administration are not suited to a program of chemiprophylaxis because children cannot finish the dose schedule and pharmacies will not use them in these cases[9].

Quinfamide, a pharmaceutical antiamoebic that acts in the intestinal lumen, is absorbed at minimal levels, and is eliminated in 48 h[10,11], has been demonstrated to be an effective clinical antiamoebic in 86 to 95% of cases involving a single day of treatment[12,13,14]. It also can be administered in one dose in a single day of treatment in children[15,16]. Because of these characteristics, we decided to use quinfamide as a chemoprophylactic agent in this study. The goals of this study were to examine whether administering quinfamide every 3 or 6 months during the year resulted in a reduction in the frequency of amoebic infection to below 27%.

## MATERIALS AND METHODS

### Type of Study

This prospective, longitudinal, randomized, single-blind study followed protocols approved by the Technical Committee for Bioethical Investigations for the School of Nursing and Obstetrics at the University of Guanajuato, Celaya. This study was also approved by Mexico's Secretary of Health. We obtained written, informed consent from the parents of all patients included in the study.

## Sampling

Primary schools in the city of Celaya that were selected for this study were Club de Leones N° 1, Librado Acevedo Ulloa, Emiliano Zapata, and Carmen Serdán. Schools selected in Neutla were José Ma. Morelos and Tierra y Libertad. The total population of these six schools was 1,708 students; of these, 1,524 were accepted for participation through approval of their parents. Faust's method of parasitoscopy by concentration (PSC)[17] was carried out on each student in a series of three analyses on consecutive days to detect *E. histolytica* cysts. Presence of cysts was identified using morphological characteristics and cellular size measured by ocular micrometry[18], and the same technique was used with each analysis. Weight and height for each patient was also taken using a scale with an altimeter, and clinical data regarding gastrointestinal conditions (abdominal pain, diarrhea without mucus, diarrhea with mucus, straining, painful stools, distension, flatulence, or alternation of diarrhea/constipation) was collected.

Patients in whom cysts were detected were included in the study. All patients received one 4.3-mg/kg dose of quinfamide taken on day 1, and PSC was performed on days 5, 6, and 7 following dosage to examine whether levels of *E. histolytica* had decreased in stools. Patients were also asked about possible adverse events and further clinical data on gastrointestinal response was collected on day 5 following administration of quinfamide. In those patients in whom *E. histolytica* cysts were detected in stools, a second dose of quinfamide at the same level as the first was adminstered and PSC was repeated on days 5, 6, and 7 following administration of the second treatment to see if the stools tested negative for *E. histolytica* cysts.

When stool samples of the participants were negative for *E. histolytica* cysts, each patient was assigned a number, and all were randomized into three groups according to a randomization table where numbers 1 through 3 were assigned to Group 1 and integrated with the 102 patients in the group; 4 through 6 were assigned to Group 2, which was made up of 98 participants; and 7 through 9 were assigned to Group 3, which consisted of 102 patients. Zero was ignored. Group 1 was used as the control group and was examined using PSC in a series of three analyses over consecutive days at the end of the 12-month study period. The quinfamide dose was adminstered as a single dose of 4.3 mg/kg on one day after they had provided stool samples. For Group 2, the three PSC analyses were performed on consecutive days at 3, 6, 9, and 12 months after intitation of the study period; each participant in this group received the 4.3-mg/kg dose of quinfamide in a single day after providing a stool sample. Group 3 underwent the three PSC analyses at 6 and 12 months after the initiation of the study period following the same dosage regimen as the other groups.

The collection of stool samples was made through home visits, and samples were immediately taken to the laboratory for processing; no more than 2 h passed between collection and lab processing for any sample. Children and their parents were also asked about any clinical gastrointestinal signs at the time the samples were collected.

## Exclusion Criteria

Patients who had received antiamoebic treatment within 15 days prior to the first evaluation were excluded, as were patients who presented with clinical signs suggesting amoebic dysentery. Also excluded were patients who reported a history of sensitivity to quinfamide.

## Administration of Medications

The medication used in the study was quinfamide, administered in doses of 4.3 mg/kg in a single dose in one day of treatment. The dose was administered by one of the study investigators to ensure compliance on the part of the participants; compliance was easily achieved because only the single dose was needed.

To maintain the single-blind nature of the study, the researcher who analyzed the stool samples was unaware of the study objectives or the identity of the study groups. The researcher who performed the sample collection and interviews was also unaware of these factors.

## Statistical Analysis

### *Sample Size*

In the control group, the frequency of amoebic infection was 27%; the frequency was 6% in the group that received medication every 3 months (95% confidence level; 80% power). The average sample size for each group was 98.

For statistical analysis, the software program EpiInfo 6.0 (Centers for Disease Control, Atlanta, GA) was used. To minimize errors in transcribing the data from the survey sheets, we entered the data twice and compared the two databases.

The mean age, weight, and height were calculated and the sex ratio for each population was determined, as well as the percentage of patients with amoebic infection. We also calculated the percentage of clinical data reported before and after the initial administration of quinfamide and analyzed these data using the $\chi^2$ test; the value of $p$ was calculated using relative risk and 95% confidence intervals[19]. The percent effectiveness of the antiamoebic quinfamide in this population was obtained, as was a percentage of adverse events in all patients who received the treatment.

The mean and standard deviations for age, weight, and height for participants for each group was calculated using the Kruskal-Wallis test[20] to evaluate the comparability between groups. The sex-based distribution for the three groups was calculated using the $\chi^2$ test and obtaining the $p$ value[19] to uncover similarities or differences between groups.

The percentages of the results of the PSC analyses for Group 2 and the percentages for clinical data collected before analysis of stool samples were also obtained. These results were compared to the PSC results for Group 1 using $\chi^2$ analysis on $2 \times 3$ contingency tables[19] to obtain $p$ values and uncover any significant differences in the results.

The same test was applied to the data on the presence of clinical symptoms (abdominal pain, diarrhea without mucus, diarrhea with mucus, straining, painful stools, distension, flatulence, or alterations of diarrhea/constipation) for the groups at the end of the 12-month period.

The presence of adverse events secondary to the administration of the quinfamide was also compared.

At the end of the study, using Group 1 as control, we compared the results of PSC at 12 months for Group 1 vs. Group 2, Group 3 vs. Group 1, and Group 2 vs. Group 3 using $\chi^2$ analysis of $2 \times 2$ contingency tables[19]. We performed the same comparisons for the clinical data and adverse events.

In all cases, the significance level for $p$ was set at 0.05.

## RESULTS

We initially evaluated 1,524 students (89.2% of the school population), and of these, 308 (20.2%) presented with *E. histolytica* cysts in their stools. Of these, six were eliminated because they did not meet the inclusion requirements (primarily because they did not provide control samples). The age range of the remaining 302 participants was from 3 to 16 years with an average of 7.97 years of age (SD = 2.5). The weight range was from 14 to 70.5 kg with an average of 29.1 kg (SD = 10.3), and the height range was 96 to 168 cm with an average of 126.1 cm (SD = 14.9). There were 132 males and 170 females in the study; thus, the female:male sex ratio in this study slightly favored females (1.3:1).

Table 1 shows the clinical data reported before and after the first treatment. Abdominal pain (73.5%), straining (53%), and diarrhea alternating with constipation (52%) were the most frequently reported before the initial administration of quinfamide; after the first administration, the most frequently reported clinical signs were the same, but the percentages were lower (22.5, 7.9, and 7.9%, respectively).

After the first quinfamide treatment, we found that 277 patients (91.7%) tested negative for *E. histolytica* cysts after PSC analysis, but that *E. histolytica* persisted in 25 participants (8.3%). After the second quinfamide treatment, 100% of the stools tested negative. There were only 13 cases of abdominal pain (4.3%), 3 cases of headache (0.99%), and 3 cases of nausea (0.99%) reported as adverse events; these cases were of low intensity, lasted less than 24 h, and did not require treatment.

Table 2 shows the demographic data for the study, divided into three groups as per the Kruskal–Wallis test. The differences in the average age and height follow the sex distribution of the groups and have no statistical significance, but the difference in average weight falls within the limit set for statistical significance.

During the study, two patients in Group 2 and one patient in Group 1 left the study because they moved out of the city.

Table 3 shows the clinical data reported at the end of the 12 months for the three groups and is presented as the result of $\chi^2$ analysis with p values. At the conclusion of the poststudy follow-up, abdominal pain was the clinical sign most frequently reported (63.4% in Group 1, 43.75% in Group 2, and 37.2% in Group 3); this frequency was followed by straining with 43.6% in Group 1, 31.2% in Group 2, and 29.4% in Group 3. For diarrhea without mucus, diarrhea with mucus, and pushing, the *p* values were greater than 0.05 (not significant), while *p* values for abdominal pain, painful stools, distension, flatulence, and diarrhea alternating with constipation were less than 0.05 (significant).

Table 4 shows the results of PSC analyses for each group after $\chi^2$ analysis to determine *p* values. The highest frequency of the presence of *E. histolytica* cysts in stools was found in Group 1 (37.6%), which received no treatment throughout the 12 months of the study. This frequency was followed by that of Group 3 (23.5%), which received treatment every 6 months (at months 6 and 12). Group 2, which received treatment four times during the study (months 3, 6, 9, and 12), had the lowest frequency of cysts (12.5%). The *p* value for this comparison was less than 0.05.

Table 5 shows the results of the Relative Risk analysis (95% confidence level). Analysis was done with $\chi^2$ tests to determine *p* values for intergroup comparisons.

As for adverse events, there were three cases of abdominal pain in Group 2 and three cases in Group 3; one case of nausea in Group 2 and one in Group 3; and one case of headache in Group 2. No participants in Group 1 reported adverse events.

**TABLE 1**
**Gastrointestinal Clinical Data Before and After* the First Quinfamide Treatment**

| | Before | | After* | | | | | |
|---|---|---|---|---|---|---|---|---|
| **Clinical Data** | **n** | **%** | **n** | **%** | **Relative Risk** | **95% CI** | $c^2$ | ***p* value** |
| Abdominal pain | 222 | 73.5 | 68 | 22.5 | 0.31 | 0.25–0.38 | 155.27 | <<0.05 |
| Diarrhea without mucus | 80 | 26.5 | 16 | 5.3 | 0.20 | 0.12–0.33 | 49.16 | <<0.05 |
| Diarrhea with mucus | 69 | 22.8 | 1 | 0.3 | 0.01 | 0.00–0.10 | 72.53 | <<0.05 |
| Straining | 160 | 53.0 | 24 | 7.9 | 0.15 | 0.10–0.22 | 142.44 | <<0.05 |
| Painful stools | 124 | 41.1 | 9 | 3.0 | 0.07 | 0.04–0.14 | 125.31 | <<0.05 |
| Distension | 132 | 43.7 | 13 | 4.3 | 0.10 | 0.06–0.17 | 126.36 | <<0.05 |
| Flatulency | 105 | 34.8 | 8 | 2.6 | 0.08 | 0.04–0.15 | 100.33 | <<0.05 |
| Alternate diarrhea/ constipation | 157 | 52.0 | 24 | 7.9 | 0.15 | 0.10–0.23 | 137.46 | <<0.05 |

* 5 days after the treatment; 95% CI = 95% confidence interval.

**TABLE 2**
**Demographics: Characteristics of the Population**

| | Group | | |
|---|---|---|---|
| | 1 | 2 | 3 |
| **Parameters** | **n = 102** | **n = 98** | **n = 102** |
| Age Mean (years) | 8.36 | 7.98 | 7.57 |
| SD | 2.18 | 2.66 | 2.58 |
| Statistical Analysis | KWH = 5.310 | df = 2 | $p = 0.07$ |
| Weight Mean (kg) | 30.28 | 29.17 | 27.89 |
| SD | 9.131 | 10.779 | 11.028 |
| Statistical Analysis | KWH = 5.874 | df = 2 | $p = 0.05$ |
| Height Mean (cm) | 128.24 | 126.31 | 123.87 |
| SD | 12.18 | 16.33 | 15.71 |
| Statistical Analysis | KWH = 4.331 | df = 2 | $p = 0.114$ |
| Gender—Male | 45 | 44 | 42 |
| Gender—Female | 57 | 60 | 57 |
| Statistical Analysis | $\chi^2 = 0.32$ | df = 2 | $p = 0.8$ |

*Note:* KWH = Kruskal–Wallis H; df = degrees of freedom; SD= standard deviation.

**TABLE 3**
**Clinical Data Reported to the End of Follow-up**

| | Groups | | | | | | | | |
|---|---|---|---|---|---|---|---|---|---|
| | 1 | | 2 | | 3 | | | | |
| **Clinical Data** | **n** | **%** | **n** | **%** | **n** | **%** | c² | **df** | ***p* value** |
| Abdominal pain | 64 | 63.37 | 42 | 43.75 | 38 | 37.25 | 14.96 | 2 | 0.0005 |
| Diarrhea without mucus | 17 | 16.83 | 14 | 14.58 | 10 | 9.8 | 2.21 | 2 | 0.33 |
| Diarrhea with mucus | 3 | 2.97 | 5 | 5.21 | 0 | 0 | 5.2 | 2 | 0.07 |
| Straining | 44 | 43.56 | 30 | 31.25 | 30 | 29.41 | 5.26 | 2 | 0.07 |
| Painful stools | 39 | 38.61 | 11 | 11.46 | 25 | 24.51 | 19.34 | 2 | 0.00006 |
| Distension | 29 | 28.71 | 11 | 11.46 | 24 | 23.53 | 9.13 | 2 | 0.01 |
| Flatulency | 34 | 33.66 | 12 | 12.5 | 23 | 23.96 | 12.44 | 2 | 0.001 |
| Alternate diarrhea/ constipation | 41 | 40.59 | 23 | 25.95 | 22 | 21.57 | 10.56 | 2 | 0.005 |

*Note*: df = degrees of freedom.

## DISCUSSION

Because of the high participation level (89.2%) from the chosen population, nonparticipation bias is not a problem. The average age of the population was expected for a group of patients chosen from

**TABLE 4**
**Positive Results, by Study Group, for *E. histolytica* Cysts in Parasitoscopic Samples**

| | Positive results | | | | | | | |
|---|---|---|---|---|---|---|---|---|
| | 3 months | | 6 months | | 9 months | | 12 months* | |
| Group and N | n | % | n | % | n | % | n | % |
| 1–n = 102 | | | | | | | 38+ | 37.62 |
| 2–n = 98 | 19+ | 19.59 | 16+ | 16.67 | 17 | 17.71 | 12 | 12.5 |
| 3–n = 102 | | | 35 | 34.31 | | | 24 | 23.53 |

* $\chi^2 = 16.8$; df = 2; $p = 0.0002$.

+ One patient missed in the follow up.

**TABLE 5**
**Intergroup Comparison of the Parasitoscopic Results**

| Compared Groups | Relative Risk | 95% CI | $c^2$ | *p* value |
|---|---|---|---|---|
| 1 vs. 2 | 0.33 | 0.18 to 0.60 | 15.10 | 0.0001 |
| 1 vs. 3 | 0.63 | 0.41 to 0.96 | 4.11 | 0.04 |
| 2 vs. 3 | 0.53 | 0.28 to 1.00 | 3.34 | 0.06 |

*Note*: 95% CI = 95% Confidence Interval.

a school-age population (in Mexico, school begins with kindergarten at age 3 and continues through primary elementary education at age 6 and upwards). There was no education on hygiene provided to participants in the study because we did not want to modify their personal cleaning habits or food consumption habits in any way so as not to bias our results. It is possible that the parents will be able to teach their children who had *E. histolytica* cysts that they can have better hygiene at school if they always wash their hands before eating and after using the bathroom.

The effectiveness of the initial treatment of quinfamide (91.7%) was similar to that reported by Padilla et al.[12,13,14,15,16,21] and Romero et al.[22]. The minimal reportage of adverse events is similar to that reported by Padilla et al.[12,13,14,15,16]; because hardly any of the quinfamide is absorbed, adverse events are kept to a minimum[10,11].

The presence of clinical gastrointestinal complaints diminished considerably after treatment, but did not disappear completely (Table 1); however, for all the clinical data reported after treatment, the difference was significant ($p \ll 0.05$), and all the clinical evaluations (95% confidence level) confirmed that the results were statistically significant. The majority of the clinical symptoms reported were similar to those reported by Padilla et al.[13] when the effects of quinfamide and nitazoxanide in children with amoebic infection, but without dysentery, were evaluated.

At the conclusion of the 12 months, we found that the differences in the results were statistically significant, with $p < 0.05$ (Table 4). In the intergroup comparisons, we found that the diminution of the of the frequency of amoebic infection was 67% for Group 2 compared to Group 1 (confidence level 95%); this level of confidence is justified in that the frequency of amoebic infection would be expected to be between 82 and 40%. The result indicates to us that there is statistical significance in the reported results ($p = 0.0001$). In the comparison of Group 1 and Group 3, we found that the diminishment of the frequency of amoebic infection would be 37% (confidence level 59 to 4%); the differences between the groups was statistically significant ($p = 0.04$). Comparing Group 2 with Group 3, we found that the diminishment of amoebic infection would be 47%, but the results indicated that the differences for this comparison were not significantly different ($p = 0.06$).

## CONCLUSION

In agreement with the results obtained in this study, a chemoprophylactic antiamoebic program should be implemented, with administration of quinfamide every 3 or 6 months. The scheme should be adapted to perform studies on populations taken from different socioeconomic levels to test whether the administration of quinfamide two or four times a year is adequate.

It would be important to carry out studies that control for personal hygiene and consumption of food outside the home in conjunction with chemoprophylaxis to examine whether such a course of treatment is a good option for the control of amoebic infection.

As long as conditions of personal hygiene, food consumption habits in the streets, and the preparation of food do not help to decrease the rate of amoebic infection in populations of schoolchildren, chemoprophylaxis with quinfamide is a good option because of its pharmaceutical characteristics, the necessity for only a single dose given on one day, the minimal adverse events, and its elevated effectiveness against amoebic infection.

## ACKNOWLEDGMENTS

This study was funded thanks to the economic support of Searle, of Mexico, S.A. of C.V. Our thanks also to Dr. Pierre Beukens, professor and head of the Department of Maternal and Child Health at the University of North Carolina at Chapel Hill, U.S.A., for his valuable comments on the final paper. We thank also Agustin Rodrigues, technical analyst, for his help in laboratory protocols, and Mercedes Jimenez and Cristina Jimenez for their help in gathering samples and conducting interviews.

## BIOSKETCH

**Nicolas Padilla** is Professor of Pediatrics and Researcher at the Nursing and Obstetrics School of Celaya, University of Guanajuato, México and Professor of Pediatrics, Microbiology, Methodology of Investigation, and Researcher at the Nursing and Obstetrics School of the Medical-Surgical Center in Celaya, Gto, México. He is also a private pediatrician. Dr. Padilla received his M.D. from the University Autonomous of Guadalajara México and his Pediatrics certification from the Mexican Council of Certification of Pediatrics. He was also a candidate to the M.Sc. Epidemiology selected by the London School of Hygiene and Medicine Tropical, University of London. His main research interest is parasitology. He is also interested in amebiasis, infections in children, congenital dislocation of hip, and epidemiology.
